# Supplementary material for: DWV 3C Protease Uncovers the Diverse Catalytic Triad in Insect RNA Viruses
Source: Microbiol Spectr. 2022 May 16;10(3):e00068-22. doi: 10.1128/spectrum.00068-22 (PMC9241925; doi:10.1128/spectrum.00068-22)
Supplement: SUPPLEMENTAL FILE 1 — Supplemental material. Download spectrum.00068-22-s001.pdf, PDF file, 0.6 MB [file spectrum.00068-22-s001.pdf]

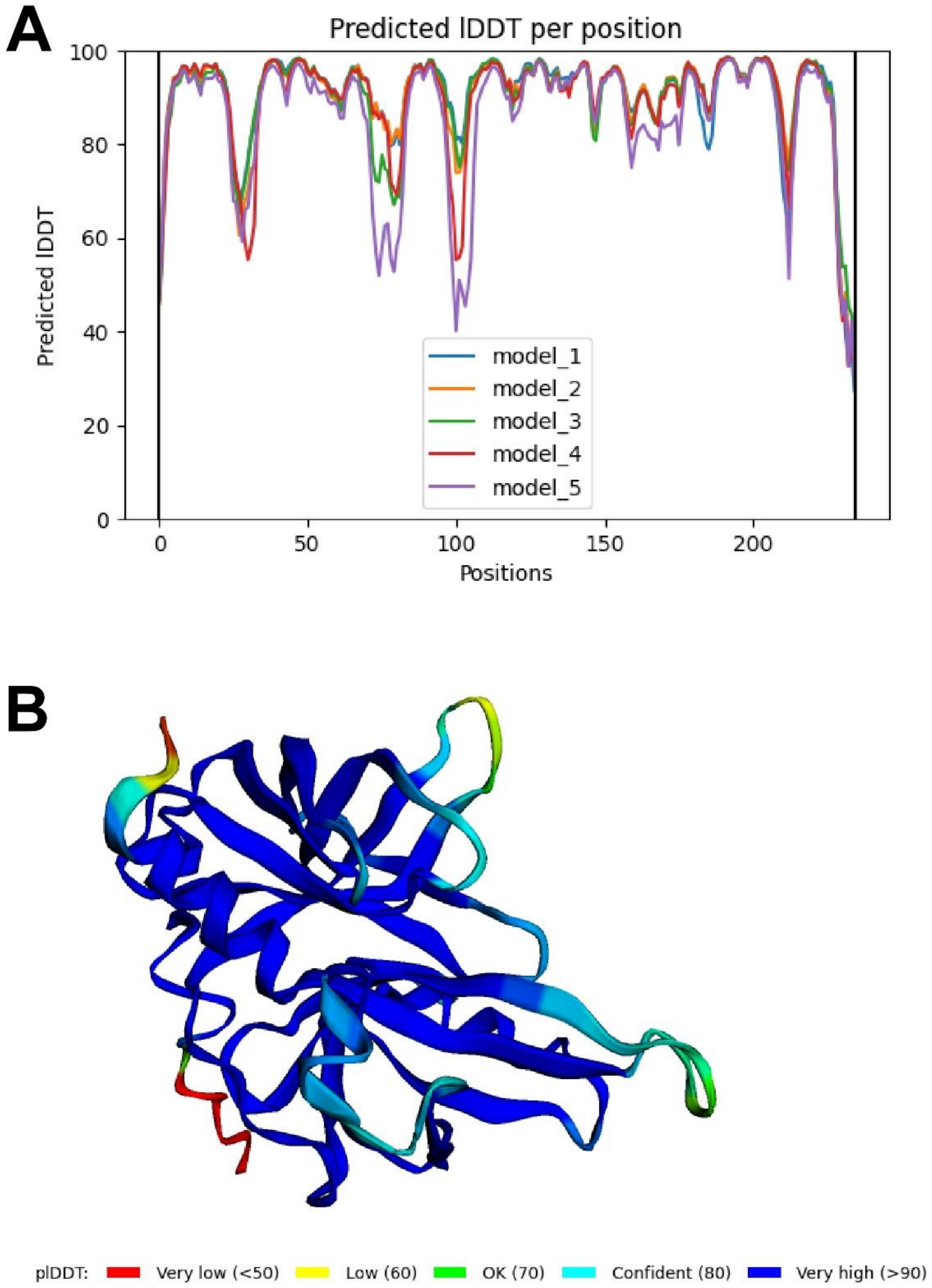

Supplementary figure 1. Confidence level of deformed wing virus (DWV) 3C proteases (3C^{pro}) structure predicted by AlphaFold2.

(A) Predicted lDDT per position shows the model confidence (out of 100) at each position of the amino acid sequence. (B) IDDT was superimposed on the predicted structure of DWV 3C^{pro}. Blue-colored region represents the predicted structure with high confidence.

```
Bemisia               ----------------------------------DAVQRKVVANTVIFTITS---DQTD-RT
Brevicoryne           -------------PQSSV-------SNNNLELISSVYNRINRNTVYI--NYED-KVLN-KI
Aedes                 --------------------------EGPKSSLDGVLNKISSNTVIVIGKYTE-DGVE-RI
Ista                  IKASQSIRAKALRLVKPEMAQRESLQEVQNQFEEKLKIIQGNTIFLIGVDPI-SGV---T
Culex                 -------------------------------------VDKILGNTVFIIARHPE-TGA---V
Hypera                ---------------------------NATNQEEVISAKIIRNTFFLYAEYGD-ETRM-TS
Watson                -----TIGAKANKKIPIAKPK---AQACAQQEDVIFKYIKRNTFWLSLEFET-ETGY-DS
Serbia                ------------------VPVKALAQSCEQQENVITKLIMKNTFWILAEYVE-HGII-KT
Rondonia              -------ACKVAKHPIARPEIAPKFQSTSQQSRELPRFLEENMVSIHADFEDERGIT-RT
Antheraea             -SKAKVTKAPKAKVRTPVRATSKLEYQSAQQFEVVQQRLKDNMSAIDVVYTNADGKD-VR
Lymantria             ------------------RATKTLEYQGAQQFDVVKQRLRNNLSSIDVVYTDVEGNM-KR
Moku                  ----YCKIDKAKPAKANFKPADKEDDGSLDRIEQLERKLLNNICFLEARWNDEKGDA-KM
Moran                 -------KSPRAGRKPDAPA----QKESAENDYYVERKLLNNVCFLEFDYINSDNKR-RC
Pityohyphantes        ---------------------------ANQNVGDVMRKISNNIVFLQLSYTW-EGIP-KS
Tribolium             -------------VKRPDVKVMSKMPEMAPQQYSNIMAAINRNTVIITAEYFNEKGSL-CS
Nilaparvata           ---------------------------SSQQSEAVIKIIRNNTFYLSICD---NTTM-RI
Erysiphe              -------------VARTAPRIGAKAQGNSQQAEYVEGLIRKNMVILRVRGTG-ELVG-RV
Psammotettix          -----TPKHPVKSKVPISSKTFVTPQAGAIQIAQLSNKITFNTVFVVVQYEC-EGRL-LE
Euscelidius           -------KFPQKPKVPTSEKVFNPPEMSTEQRQVMINRINKNGVLIYVNWVQ-DGTR-MN
Formica               -NLRYDYQTPIKNPPKHIPVAFAKPQSNLPQIEVVTKLIHRNTFFLHCYIDD----R-HI
King                  --------APKAGVKPVVS------QSESQQYEVVLKLIRSNFFYLTMV-----ANG-KV
Pittosporum           -------------------------QAMMPQYNACIDRINKNSCTVVCYYGV--NSD-KM
Darwin                -GLKYGEHVTPKARLSPRNVPSAKPQDDTQQVDVALKRIRANMCYIECHRPD----G-YT
DWV                   -GLKYSEAVTVKAPRIHRLPVTTKPQGSTQQVDAAVNKILQNMVYIGVVFPKVPGSKWRD
                                                            :  *   .

Bemisia               ARCFGLGIYNRFVLLPKHYKLTFLNL-----TTEKLYCEPFGERHLRQHIDPT-------
Brevicoryne           SHMKCIVIKERYVIVLRHYIEYLECNAT---DTSNVNITWADCGSY-----------PFN
Aedes                 ARARCLALHTRTLLVLRHYFEFFKYK-----NVEKVTVVSRKGDCSV-EYGLDEL--QFM
Ista                  YKGRCLGLYDRTVLAIKHYFDHFLHK-----GIKRLKVVRPNSNTTIYDVDIDNL--KVQ
Culex                 YKGRCLGLYGKEVLVVKHYFDLFRKI-----GVKKVEIVRPRVEATIFTVDISDL--LFA
Hypera                RVFRCLGLNRHNFIIIDHYVTFLRQQ-----PNLKLSFVRDGCCVEISTA----------
Watson                RLFRCLGLVGKYFLFLDHYYNFMKSKP----NAKLFYIQQGMYIEIPLSVVNQNI-----
Serbia                KILRCLGLYKHYFLLLDHYIGALRMMD----DLKLSFIKQGTYITIPNSVVFTNI-----
Rondonia              SVSSCFVICDRRMLILRHYWDHWIKLP----PTTKFYLVSRKTVSVNY---PKGI--PLT
Antheraea             VRNYGLMLRDQQMLIQKHYYDFWRRLD----VTAKFYF----CNQKFKSIHPDGI--IIS
Lymantria             TRNFGLMLKDQQMLIQKHYYDFWKRLD----LTAKFYFYNNNIKSHA----PDGI--LLT
Moku                  LYGRCLGIRERQVLILKHYIEEMLHIP---SDATFMINYIMSGKPCT----------GFL
Moran                 ITGRCLGIRNREIVVIKHYLEEMKSLAKKYPEHGLTIVLCRNELDSRIKLTMEDLD-KVS
Pityohyphantes        KKFRVVMLRERQCLMIRHYIEDIQFYADHD-PNAKLSLLVNGNNEVPLAINCVN---EFS
Tribolium             MKARALGLEANRVIMIRHYHDEFTAMP----PSTRYYVSLVTNNQLRPRV-------EIN
Nilaparvata           YRYRCLGIHGHFALMLRHYVDNIKDKIRCV-GTTNLTIAVEYNHNCVLTG-SRTI--QLD
Erysiphe              WNFVCICLGDRNILALRHYLDVIDRYS----NSVTLSLAYAERHDSRLKV-LDGV--EML
Psammotettix          KSCRCLMLRGRAMLILRHYWEEYQYLVD---RGYKLDVALHFGAGRK-SAEPRHVIRRVG
Euscelidius           RNCRCLMLGGRNMLVLRHYLEEYSALVE---QGFKLNVDLVF--GNK-IGDPVKV--NIT
Formica               VAMRGVVMANREALVLRHYIEQIKGLQERYKEHLKITFTWHQQQYERMNC--KEL--EID
King                  VTYRCLRLRMNEVLILRHYDDEIR-------SVGDVAVTYGKCAGLNADTFKYGI--QFN
Pittosporum           IHFRCLILRNREMLFLRHYREQVVNL-----PNAQFRFKFNVDNEKR-TGNPDGI--PFD
Darwin                KRYRALLLKNRMMLFLRHYHENIMKQP----EGTLVIFTYAFNSEERIKC-VRGI--ELR
DWV                   INFRCLMLHNRQCLMLRHYIESTAAFP----EGTKYYFKYIHNQETRMSGDISGI--EID
                           . :  .  :   **
```

```
Bemisia          -WL-VY--------------------EDIEDADLCILRLPANFP-MFKDIRKYMATMHDH
Brevicoryne      -YK-DY--KIYWCE---------------NSN-IGVLRLGNWFS-ARRSLIPFITKSDSF
Aedes            -WSDDC--------------------G------YGTCELPQSYPVNFKKITQLICSDKFS
Ista             -WG-DC--------------------G------YGLLYLPQSYPTQFRKITQFFPSEEQT
Culex            -WG-DC--------------------G------YGMMTLPRSFPTQFKKITQLIASEELN
Hypera           -FLSEC--------------------KAIKDSALMVAEMDKTVP-QFVNIIKFFIRSNQT
Watson           --------------------------QTLTNSTFMIGKMHKTIP-HFANIIKLMIKQEAS
Serbia           --------------------------KKLQNSALCIGTMYKTIP-MFSNIIKFIIKAGDT
Rondonia         -LG-NL--QPEWFADIDSS-------GFYKSH-FGIAQLPQSSP-AFKDLRKFIATVEDH
Antheraea        NLF-DL--DVDWFMTPGL--------EYYDSN-FGVLHLPKTVP-AFKDITRFIAKSSEH
Lymantria        NFF-DL--DVDWFMTPDQ--------DIFDSN-FGILHLPKIVP-AYKDLTKFIAKSTEH
Moku             -TR-KCLESVTYFSIN----------GKINASNYGILTLPKFMP-MFKDILNSIVRKNDH
Moran            -WM-KIG-------------------NEENTSNFGILTLPIRVP-QFRNIVNSIATIGQH
Pityohyphantes   -VL-ENQIETDFDEEREGT-------GYVLYNGLCVVDLPKTVR-EFPTITRHFVSAKDE
Tribolium        -YR-ECRCDAFYCKD-----------PKYGTN-FFMLYMPPHIP-LFRNIKNLIPTAESH
Nilaparvata      -ND-AFLASCKFFKHKAPEFYSEDQFNAHQSN-FVVFKVPSQCL-SFKSLLKFFPSQAEV
Erysiphe         -YE-NLHRQEYFYDE-----------GKIDSN-FVVFQAPARFQ-PMRNLMKFIASGQEH
Psammotettix     -WA-EL-QKVAWCSS--SS-------GELTSN-FGICELPMYIP-MFCDITNYIASMSEH
Euscelidius      -FT-ELMSQVAYCDN---------------SN-FCIVVLPKFVR-QFPKIYPYFATRANH
Formica          -FL-NC--RIIYYGSTDLS-------GCAMSN-LCIVELPARIP-EXXXLKKFVCAEASH
King             -YR-EC--KIKWYRNVVVD-------GCYPSN-FGILLLPPCFP-MAKDLTKFIATSDDH
Pittosporum      -FL-GC--KTSYYGSVE---------NCMFTSNLGIMVLPVNIP-ECRNITKFIMPLNKH
Darwin           -YQ-DL--EKTEYG---RQ-------DQYDSN-FMIVKLPASIP-EAKDLTKFISPIDEH
DWV              -LL-NL--PRLYYGGLAGE-------ESFDSN-IVLVTMPNRIP-ECKSIIKFIASHNEH
                                                                 :   .

Bemisia          EVRGLPRQAYILSPPIRKAPHDIFKEIAVNISGYY------D-DLSTKCDGVQIVT---K
Brevicoryne      LGNAGRECVILDVK------LSESYVVYNENIKLVD------N-VTIAPTSYSKMLIM--P
Aedes            RCYP-SNMMIVEHN------FSNLHTLEVQAKVMS------ESKKIPQQPGMSSWVI--H
Ista             SEYP-ASVQMFEDS------GAGYKLFSMEMTVID------E-VVVPKSGTFEAWTI--S
Culex            TEYP-AAIQIVEDK------GVGHTLISTNMRLIQ------E-QVVPANQEFEAWTI--F
Hypera           GNLS-PLATLHEYVWDANKQNYSLRSRDFSRISRQ------N-CVSVTNDDDSRTIV--S
Watson           GNIA-PQAKLYEYK-LNSDREFMMQIHHIDRIYRQ------D-RLEVNNDDGSVSRI--G
Serbia           KNIC-PQAKLYEYS--VEGESYVMKVQSFARIMRK------D-QVDVSNDDNTVTHV--S
Rondonia         RYIDTQACYLYDSQ--------KRCQVILPIQLQK------G-YIVSDGDNGTHLPI---
Antheraea        SYIKFDECYLYSSL------TDMCMHCVMNVEMNK------D---VTDSNGWLQL----S
Lymantria        QYIKFDECYLYSSL------SGESMHCVMNIEYNK------E---VTDANGWLRL----D
Moku             HYVA-SQGHIMSSD----MEGRILRHRHMQLRPRA------F-LVIDGDDEGHVSTICND
Moran            LNVR-SVGDFLSHK------GKTRRRIVIQEKRV---------LVVSKTENTTGAIL--D
Pityohyphantes   LRMS-NNGILLMPEPGKGSLANTIVCENVKFSNYE------N-LTVNAADTTSAVHL--E
Tribolium        QYCG-RLGHLYAFG------DATYADLAFNFEG--------E-YRIQQWGNAPGMVC--M
Nilaparvata      NQAS-TTLRLVNEK------DN--MELYGKLKSHKVGGLYIP-PVHKENAYNDAVTM--A
Erysiphe         TYIS-QKCMFIRPN------QLATEVCVETRNMPV--------IIEPACEGAAEVHL--V
Psammotettix     ENVS-SLCDLYVVN------GE--SKFGMPLSVKR------R-FAVAETASSSQVKV--D
Euscelidius      DNVS-GKVDMITVA------GE--SSFDLPVSVSK------N-FVVSETANSSEVVC--E
Formica          CRAG-TTGRLIEPG------THNILQLKIDYNSKM------P-YVIEPYGDTKQVNM--L
King             KYSS-GFGSLVEGD-------KVVEHMRITRDSKR------P-FVIPKTDIVSPVAM--D
Pittosporum      RYAS-TTGSLVQPG------GNTLNQVTIDYSNKQ------P-HTIAGMNGSTDVYM--Q
Darwin           RRTL-SDGILVNEK------NTELVHFS---PQNR------P-HIIAEGD-LRDVIM--E
DWV              IRAQ-NDGVLVTGD------HTQLLAFE---NNNK------T-PISINADGLYEVIL--Q
```

```
Bemisia             EAIQYNY-SQPGMCGAILLSRNTQR---PILGMHVAGTCVDF-GFQGMGFSAILVQEIF-
Brevicoryne         NAYMYKK-NYPGMCGSVLMNEATNT---PILGIHVAGAN----GK---GFSEPICRAQF-
Aedes               DGFEYAW-GGKGKCGSLLLVPSLSS---PIVGIHSAGVKD---VR---GFAEVLFRETF-
Ista                NGFKYPW-GGSGRCGSLLLSPKLAS---PIIGIHTAGTD----GRI--GYAERLLRETF-
Culex               DGFKYDW-GGSGCCGSLILSPTLSS---PIIGIHTAGIGK---TT---GFGERLYRESFD
Hypera              CLYEYPY-GGKGVCGSVLVSRQPLT--NPILGIHIAGFSD---GSR--GYSEAIVYETF-
Watson              ANYEYPV-SGRGVCGSVLVSSSNMN--APILGIHVAGIKN---GIE--GYAEALVYESFS
Serbia              SLYAYPY-AHRGVCGSVLVSTSNMN--APILGIHIAGFSN---GVE--GFAEAITYETF-
Rondonia            -AYKYRY-TGDGKCGSILVCGNLQR---PIIGMHFAGSN----IW---GSSEPIYSENFK
Antheraea           ECYSYKY-SAKGLCGSALLCATLER---PIIGIHFAGTK----VF---GYAEPIAYETF-
Lymantria           ECYSYKY-TGVGLCGSALLCSTLER---PIIGVHFAGTS----TY---GYAEPLCYESF-
Moku                GAYEYSV-HGKGMCGSLLISDKVCRGNPGIIGLHVAGSK----GN---GYAEPIYREMF-
Moran               MAYQYGH-HGQGLCGSLLVCENVCNGNIGIIGMHVAGRS----GI---GYSEPLCREWFT
Pityohyphantes      RVWMYRGVHGYGLCGSLLLNEDSGK----IIGIHTAGSSTN--NV---GFSERLVYEEW-
Tribolium           NAYTYSK-HKPGLCGSVLISEGLPS--TPLIGMHVAGAN----GK---GYAEPLFREMF-
Nilaparvata         SYWEYGV-HGRGMCGSVLVSNNLQN---PIVGMHVAGCDNA--IK---GYSELIVKEMF-
Erysiphe            EVYKYPKMQREGFCGSIIICPTLQC---PIIGMHVAGHSEGM-GY---GIAEPLVKEMF-
Psammotettix        RAYQYNH-QYKGLCGSVLVSRKGNSGNGSIIGIHVAGSQSS--GN---GFSEPIVREYLD
Euscelidius         RVYGYAK-RSPGLCGSVIISNSLGSGNGAIIGMHIAGNAAS--GT---GYAEPLYQEMFA
Formica             NVYTYDY-HAQGACGSILLADNLEQ---PIVGIHVAGTVGKGCGR---GVAEPLSAEMM-
King                DVYVYTK-HGAGVCGSVLLAANMQR---PIIGMHVAGYGTVS-GN---GLAEILCSEMFS
Pittosporum         TVYTYQY-HAPGMCGSVLLSSGSET---PIIGIHVAGYEGSGMSY---GMSEILHREMFD
Darwin              TSYTYNY-HGRGVCGSVLLSRNLQK---PIIGMHVAGVEGVN-GY---GISEPLFYETFV
DWV                 GVYTYPY-HGDGVCGSILLSRNLQR---PIIGIHVAGTEGLH-GF---GVAEPLVHEMFT
                        *      * **: ::          ::*:* **           * .  :

Bemisia             --------
Brevicoryne         --------
Aedes               --------
Ista                --------
Culex               ADSESE--
Hypera              --------
Watson              --------
Serbia              --------
Rondonia            RSVEKE--
Antheraea           --------
Lymantria           --------
Moku                --------
Moran               G-------
Pityohyphantes      --------
Tribolium           --------
Nilaparvata         --------
Erysiphe            --------
Psammotettix        G-------
Euscelidius         --------
Formica             --------
King                --------
Pittosporum         NMPLPEE-
Darwin              SVPKSIDT
DWV                 GKAIESE-
```

>DWV
GLKYSEAVTVKAPRIHRLPVTTKPQGSTQQVDAAVNKILQNMVYIGVVFPKVPGSKWRDINFRCLMLHNRQCLMLRHYIE
STAAFPEGTKYYFKYIHNQETRMSGDISGIEIDLLNLPRLYYGGLAGEESFDSNIVLVTMPNRIPECKSIIKFIASHNEH
IRAQNDGVLVTGDHTQLLAFENNNKTPISINADGLYEVILQGVYTYPYHGDGVCGSILLSRNLQRPIIGIHVAGTEGLHG
FGVAEPLVHEMFTGKAIESE

>QCL11100.1:2261-2493 polyprotein [King virus]
APKAGVKPVVSQSESQQYEVVLKLIRSNFFYLTMVANGKVVTYRCLRLRMNEVLILRHYDDEIRSVGDVAVTYGKCAGLN
ADTFKYGIQFNYRECKIKWYRNVVVDGCYPSNFGILLLPPCFPMAKDLTKFIATSDDHKYSSGFGSLVEGDKVVEHMRIT
RDSKRPFVIPKTDIVSPVAMDDVYVYTKHGAGVCGSVLLAANMQRPIIGMHVAGYGTVSGNGLAEILCSEMFS

>AWK77848.1:2012-2262 polyprotein [Darwin bee virus 3]
GLKYGEHVTPKARLSPRNVPSAKPQDDTQQVDVALKRIRANMCYIECHRPDGYTKRYRALLLKNRMMLFLRHYHENIMKQ
PEGTLVIFTYAFNSEERIKCVRGIELRYQDLEKTEYGRQDQYDSNFMIVKLPASIPEAKDLTKFISPIDEHRRTLSDGIL
VNEKNTELVHFSPQNRPHIIAEGDLRDVIMETSYTYNYHGRGVCGSVLLSRNLQKPIIGMHVAGVEGVNGYGISEPLFYE
TFVSVPKSIDT

>QED21536.1:2477-2716 polyprotein [Moran virus]
KSPRAGRKPDAPAQKESAENDYYVERKLLNNVCFLEFDYINSDNKRRCITGRCLGIRNREIVVIKHYLEEMKSLAKKYPE
HGLTIVLCRNELDSRIKLTMEDLDKVSWMKIGNEENTSNFGILTLPIRVPQFRNIVNSIATIGQHLNVRSVGDFLSHKGK
TRRRIVIQEKRVLVVSKTENTTGAILDMAYQYGHHGQGLCGSLLVCENVCNGNIGIIGMHVAGRSGIGYSEPLCREWFTG

>YP_009351892.1:2274-2510 polyprotein [Pityohyphantes rubrofasciatus iflavirus]
ANQNVGDVMRKISNNIVFLQLSYTWEGIPKSKKFRVVMLRERQCLMIRHYIEDIQFYADHDPNAKLSLLVNGNNEVPLAI
NCVNEFSVLENQIETDFDEEREGTGYVLYNGLCVVDLPKTVREFPTITRHFVSAKDELRMSNNGILLMPEPGKGSLANTI
VCENVKFSNYENLTVNAADTTSAVHLERVWMYRGVHGYGLCGSLLLNEDSGKIIGIHTAGSSTNNVGFSERLVYEEW

>QNS17457.1:2158-2380 RNA-dependent RNA polymerase [Serbia picorna-like virus 2]
VPVKALAQSCEQQENVITKLIMKNTFWILAEYVEHGIIKTKILRCLGLYKHYFLLLDHYIGALRMMDDLKLSFIKQGTYI
TIPNSVVFTNIKKLQNSALCIGTMYKTIPMFSNIIKFIIKAGDTKNICPQAKLYEYSVEGESYVMKVQSFARIMRKDQVD
VSNDDNTVTHVSSLYAYPYAHRGVCGSVLVSTSNMNAPILGIHIAGFSNGVEGFAEAITYETF

>QKK82957.1:1131-1364 hypothetical protein [Pittosporum tobira picorna-like virus]
QAMMPQYNACIDRINKNSCTVVCYYGVNSDKMIHFRCLILRNREMLFLRHYREQVVNLPNAQFRFKFNVDNEKRTGNPDG
IPFDFLGCKTSYYGSVENCMFTSNLGIMVLPVNIPECRNITKFIMPLNKHRYASTTGSLVQPGGNTLNQVTIDYSNKQPH
TIAGMNGSTDVYMQTVYTYQYHAPGMCGSVLLSSGSETPIIGIHVAGYEGSGMSYGMSEILHREMFDNMPLPEE

>QHD64830.1:2097-2334 RdRp [Erysiphe necator associated picorna-like virus 1]
VARTAPRIGAKAQGNSQQAEYVEGLIRKNMVILRVRGTGELVGRVWNFVCICLGDRNILALRHYLDVIDRYSNSVTLSLA
YAERHDSRLKVLDGVEMLYENLHRQEYFYDEGKIDSNFVVFQAPARFQPMRNLMKFIASGQEHTYISQKCMFIRPNQLAT
EVCVETRNMPVIIEPACEGAAEVHLVEVYKYPKMQREGFCGSIIICPTLQCPIIGMHVAGHSEGMGYGIAEPLVKEMF

>QQL13637.1:2182-2425 polyprotein [Antheraea mylitta iflavirus]
SKAKVTKAPKAKVRTPVRATSKLEYQSAQQFEVVQQRLKDNMSAIDVVYTNADGKDVRVRNYGLMLRDQQMLIQKHYYDF
WRRLDVTAKFYFCNQKFKSIHPDGIIISNLFDLDVDWFMTPGLEYYDSNFGVLHLPKTVPAFKDITRFIAKSSEHSYIKF
DECYLYSSLTDMCMHCVMNVEMNKDVTDSNGWLQLSECYSYKYSAKGLCGSALLCATLERPIIGIHFAGTKVFGYAEPIA
YETF

>YP_009047245.1:2212-2438 polyprotein [Lymantria dispar iflavirus 1]
RATKTLEYQGAQQFDVVKQRLRNNLSSIDVVYTDVEGNMKRTRNFGLMLKDQQMLIQKHYYDFWKRLDLTAKFYFYNNNI
KSHAPDGILLTNFFDLDVDWFMTPDQDIFDSNFGILHLPKIVPAYKDLTKFIAKSTEHQYIKFDECYLYSSLSGESMHCV
MNIEYNKEVTDANGWLRLDECYSYKYTGVGLCGSALLCSTLERPIIGVHFAGTSTYGYAEPLCYESF

>QHI42120.1:2186-2431 polyprotein [Rondonia iflavirus 1]
ACKVAKHPIARPEIAPKFQSTSQQSRELPRFLEENMVSIHADFEDERGITRTSVSSCFVICDRRMLILRHYWDHWIKLPP
TTKFYLVSRKTVSVNYPKGIPLTLGNLQPEWFADIDSSGFYKSHFGIAQLPQSSPAFKDLRKFIATVEDHRYIDTQACYL
YDSQKRCQVILPIQLQKGYIVSDGDNGTHLPIAYKYRYTGDGKCGSILVCGNLQRPIIGMHFAGSNIWGSSEPIYSENFK
RSVEKE

>YP_009305421.1:2255-2502 polyprotein [Moku virus]
YCKIDKAKPAKANFKPADKEDDGSLDRIEQLERKLLNNICFLEARWNDEKGDAKMLYGRCLGIRERQVLILKHYIEEMLH
IPSDATFMINYIMSGKPCTGFLTRKCLESVTYFSINGKINASNYGILTLPKFMPMFKDILNSIVRKNDHHYVASQGHIMS
SDMEGRILRHRHMQLRPRAFLVIDGDDEGHVSTICNDGAYEYSVHGKGMCGSLLISDKVCRGNPGIIGLHVAGSKGNGYA
EPIYREMF

>YP_009328891.1:2309-2548 polyprotein [Euscelidius variegatus virus 1]
KFPQKPKVPTSEKVFNPPEMSTEQRQVMINRINKNGVLIYVNWVQDGTRMNRNCRCLMLGGRNMLVLRHYLEEYSALVEQ
GFKLNVDLVFGNKIGDPVKVNITFTELMSQVAYCDNSNFCIVVLPKFVRQFPKIYPYFATRANHDNVSGKVDMITVAGES
SFDLPVSVSKNFVVSETANSSEVVCERVYGYAKRSPGLCGSVIISNSLGSGNGAIIGMHIAGNAASGTGYAEPLYQEMFA

>AUE23905.1:2261-2492 polyprotein, partial [Tribolium castaneum iflavirus]
VKRPDVKVMSKMPEMAPQQYSNIMAAINRNTVIITAEYFNEKGSLCSMKARALGLEANRVIMIRHYHDEFTAMPPSTRYY
VSLVTNNQLRPRVEINYRECRCDAFYCKDPKYGTNFFMLYMPPHIPLFRNIKNLIPTAESHQYCGRLGHLYAFGDATYAD
LAFNFEGEYRIQQWGNAPGMVCMNAYTYSKHKPGLCGSVLISEGLPSTPLIGMHVAGANGKGYAEPLFREMF

>YP_008130310.1:2380-2618 polyprotein [Nilaparvata lugens honeydew virus-3]
SSQQSEAVIKIIRNNTFYLSICDNTTMRIYRYRCLGIHGHFALMLRHYVDNIKDKIRCVGTTNLTIAVEYNHNCVLTGSR
TIQLDNDAFLASCKFFKHKAPEFYSEDQFNAHQSNFVVFKVPSQCLSFKSLLKFFPSQAEVNQASTTLRLVNEKDNMELY
GKLKSHKVGGLYIPPVHKENAYNDAVTMASYWEYGVHGRGMCGSVLVSNNLQNPIVGMHVAGCDNAIKGYSELIVKEMF

>YP_009553259.1:2363-2614 polyprotein [Psammotettix alienus iflavirus 1]
TPKHPVKSKVPISSKTFVTPQAGAIQIAQLSNKITFNTVFVVVQYECEGRLLEKSCRCLMLRGRAMLILRHYWEEYQYLV
DRGYKLDVALHFGAGRKSAEPRHVIRRVGWAELQKVAWCSSSSGELTSNFGICELPMYIPMFCDITNYIASMSEHENVSS
LCDLYVVNGESKFGMPLSVKRRFAVAETASSSQVKVDRAYQYNHQYKGLCGSVLVSRKGNSGNGSIIGIHVAGSQSSGNG
FSEPIVREYLDG

>QGA70909.1:2053-2286 RNA-dependent RNA polymerase [Ista virus]
IKASQSIRAKALRLVKPEMAQRESLQEVQNQFEEKLKIIQGNTIFLIGVDPISGVTYKGRCLGLYDRTVLAIKHYFDHFL
HKGIKRLKVVRPNSNTTIYDVDIDNLKVQWGDCGYGLLYLPQSYPTQFRKITQFFPSEEQTSEYPASVQMFEDSGAGYKL
FSMEMTVIDEVVVPKSGTFEAWTISNGFKYPWGGSGRCGSLLLSPKLASPIIGIHTAGTDGRIGYAERLLRETF

>QWC36469.1:1934-2146 polyprotein [Bemisia tabaci ifla-like virus 1]
DAVQRKVVANTVIFTITSDQTDRTARCFGLGIYNRFVLLPKHYKLTFLNLTTEKLYCEPFGERHLRQHIDPTWLVYEDIE
DADLCILRLPANFPMFKDIRKYMATMHDHEVRGLPRQAYILSPPIRKAPHDIFKEIAVNISGYYDDLSTKCDGVQIVTKE
AIQYNYSQPGMCGAILLSRNTQRPILGMHVAGTCVDFGFQGMGFSAILVQEIF

>QED21508.1:2165-2399 polyprotein [Watson virus]
TIGAKANKKIPIAKPKAQACAQQEDVIFKYIKRNTFWLSLEFETETGYDSRLFRCLGLVGKYFLFLDHYYNFMKSKPNAK
LFYIQQGMYIEIPLSVVNQNIQTLTNSTFMIGKMHKTIPHFANIIKLMIKQEASGNIAPQAKLYEYKLNSDREFMMQIHH
IDRIYRQDRLEVNNDDGSVSRIGANYEYPVSGRGVCGSVLVSSSNMNAPILGIHVAGIKNGIEGYAEALVYESFS

>QUS52852.1:2169-2384 polyprotein [Hypera postica associated iflavirus 1]
NATNQEEVISAKIIRNTFFLYAEYGDETRMTSRVFRCLGLNRHNFIIIDHYVTFLRQQPNLKLSFVRDGCCVEISTAFLS
ECKAIKDSALMVAEMDKTVPQFVNIIKFFIRSNQTGNLSPLATLHEYVWDANKQNYSLRSRDFSRISRQNCVSVTNDDDS
RTIVSCLYEYPYGGKGVCGSVLVSRQPLTNPILGIHIAGFSDGSRGYSEAIVYETF

>QRW42579.1:2196-2401 polyprotein [Culex Iflavi-like virus 3]
VDKILGNTVFIIARHPETGAVYKGRCLGLYGKEVLVVKHYFDLFRKIGVKKVEIVRPRVEATIFTVDISDLLFAWGDCGY
GMMTLPRSFPTQFKKITQLIASEELNTEYPAAIQIVEDKGVGHTLISTNMRLIQEQVVPANQEFEAWTIFDGFKYDWGGS
GCCGSLILSPTLSSPIIGIHTAGIGKTTGFGERLYRESFDADSESE

>YP_001285409.1:2210-2424 polyprotein [Brevicoryne brassicae virus - UK]
PQSSVSNNNLELISSVYNRINRNTVYINYEDKVLNKISHMKCIVIKERYVIVLRHYIEYLECNATDTSNVNITWADCGSY
PFNYKDYKIYWCENSNIGVLRLGNWFSARRSLIPFITKSDSFLGNAGRECVILDVKLSESYVYNENIKLVDNVTIAPTSY
SKMLIMPNAYMYKKNYPGMCGSVLMNEATNTPILGIHVAGANGKGFSEPICRAQF

>QGW51140.1:2015-2226 polyprotein [Aedes vexans iflavirus]
EGPKSSLDGVLNKISSNTVIVIGKYTEDGVERIARARCLALHTRTLLVLRHYFEFFKYKNVEKVTVVSRKGDCSVEYGLD
ELQFMWSDDCGYGTCELPQSYPVNFKKITQLICSDKFSRCYPSNMMIVEHNFSNLHTLEVQAKVMSESKKIPQQPGMSSW
VIHDGFEYAWGGKGKCGSLLLVPSLSSPIVGIHSAGVKDVRGFAEVLFRETF

>AWI42879.1:2083-2335 polyprotein, partial [Formica cinerea virus 2]
NLRYDYQTPIKNPPKHIPVAFAKPQSNLPQIEVVTKLIHRNTFFLHCYIDDRHIVAMRGVVMANREALVLRHYIEQIKGL
QERYKEHLKITFTWHQQQYERMNCKELEIDFLNCRIIYYGSTDLSGCAMSNLCIVELPARIPEXXXLKKFVCAEASHCRA
GTTGRLIEPGTHNILQLKIDYNSKMPYVIEPYGDTKQVNMLNVYTYDYHAQGACGSILLADNLEQPIVGIHVAGTVGKGC
GRGVAEPLSAEMM

Supplementary figure 2. Alignment of the amino acid sequences of Iflavirus 3C proteases (3C^{pro}).

Amino acid sequences of 24 Iflavirus 3C^{pro}s, including DWV 3C^{pro}, were aligned using Multiple Sequence Comparison by Log-Expectation (MUSCLE). The conserved amino acids are indicated by asterisks, and similar amino acids are shown by either a period (.) or colon (:). The catalytic triad (C2307, H2170, and N2227) is highlighted in green, and the conserved amino acids substituted with alanine are shown in yellow. N2227 is not well conserved between Iflaviruses as highlighted in light blue. The self-cleavage site of DWV 3C^{pro}, Q2118, is indicated in purple. The accession numbers and aligned sequences of each virus are shown after the aligned sequences.

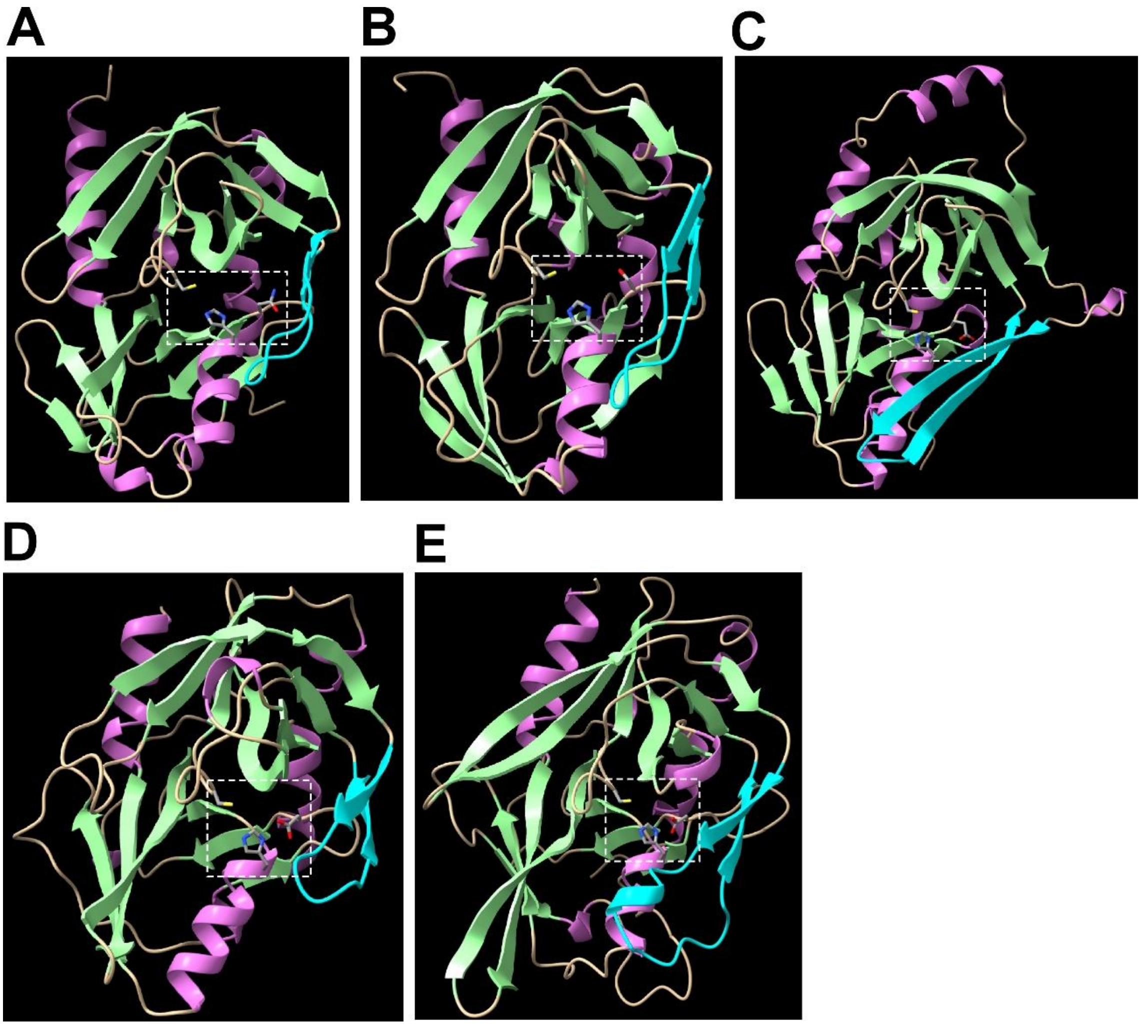

Supplementary figure 3. The 3C protease (3C^{pro}) structures of four Iflaviruses and Dicistrovirus predicted by AlphaFold2.

The 3C^{pro} structures of *Brevicoryne brassicae* virus (A), *Laodelphax striatellus* picorna-like virus 2 (B), *Spodoptera exigua* iflavirus (C), Sacbrood virus (D), and Cricket paralysis virus (E). The helix, strand, and coil structures are indicated by orchid, pale green, and wheat colors, respectively. β-ribbons are colored by cyan. The catalytic triad with amino acid residues is indicated by a square with a white dotted line.

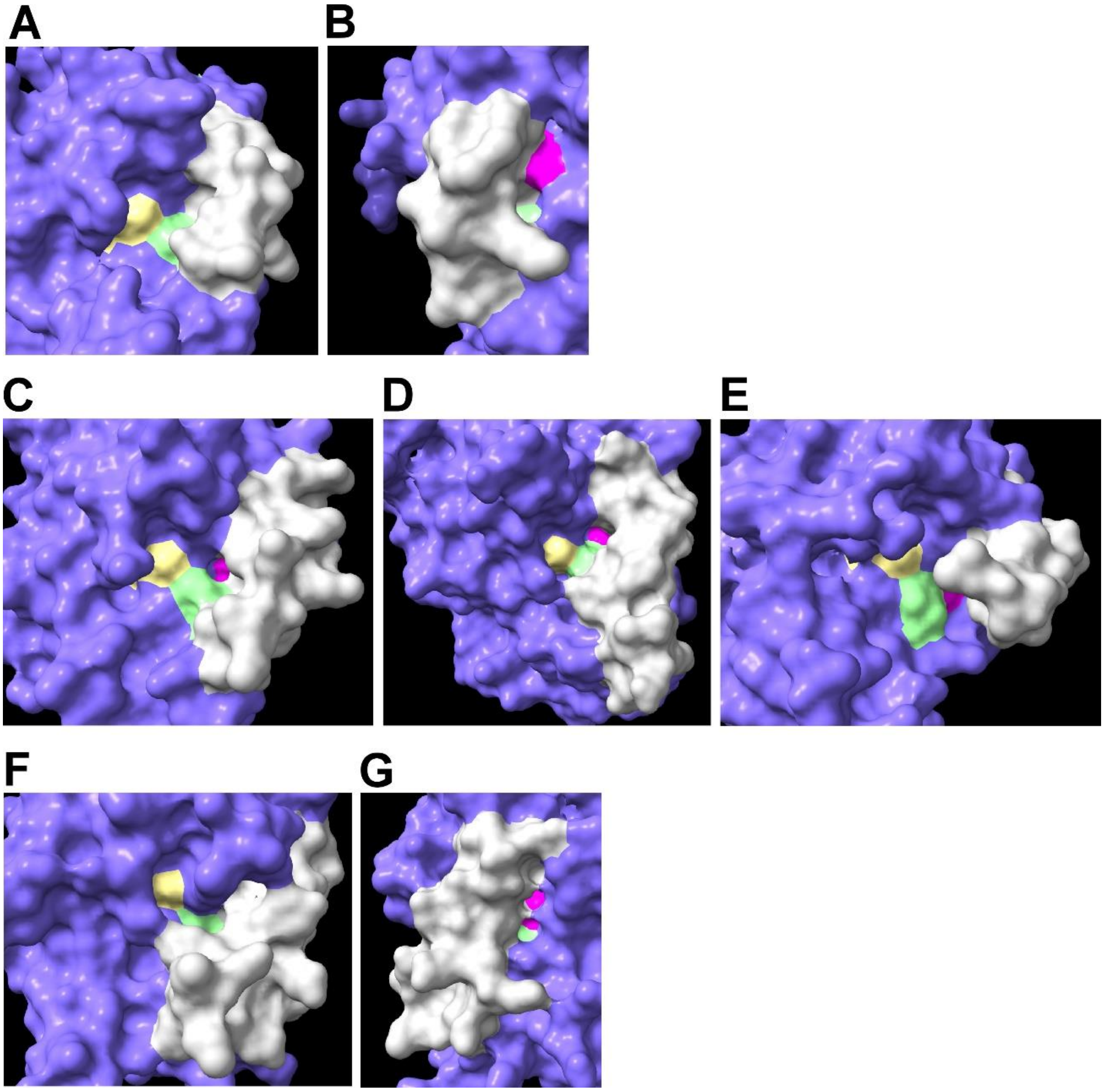

Supplementary figure 4. Surface views of the 3C proteases (3C^{pro}) of four Iflaviruses and Dicistrovirus.

Surface views of the 3C^{pro}s of *Brevicoryne brassicae* virus (A and B), *Laodelphax striatellus* picorna-like virus 2 (C), *Spodoptera exigua* iflavirus (D), sacbrood virus (E), and cricket paralysis virus (F and G). Images B and G were obtained by rotating images A and F at 60° and 90°, respectively. Cys, His, and Asn/Ser/Glu/Asp residues in the predicted catalytic triad are indicated by khaki, pale green, and magenta colors, respectively. β-ribbons are highlighted in silver.

Supplementary Table 1 List of primers used in this study

GST-3C^{pro}-5-BamHI	TTTGGATCCGGATTGAAATATAGTGAAGCAGT
GST-3C^{pro}-3-NotI	TTTGCGGCCGCTTCACTCTCGATTGCTTTACC
pGEX 5' primer	GGGCTGGCAAGCCACGTTTGGTG
pGEX 3' primer	CCGGGAGCTGCATGTGTCAGAGG
N2134A-For	AATAAAATTTTACAGGCGATGGTTTACATTGGT
N2134A-Rev	ACCAATGTAAACCATCGCCTGTAAAATTTTATT
R2156A-For	CGAGATATTAATTTTGCGTGTCTTATGCTTCAT
R2156A-Rev	ATGAAGCATAAGACACGCAAAATTAATATCTCG
L2168A-For	AGGCAATGTTTAATGGCGAGGCATTATCTCGAG
L2168A-Rev	CTCGAGATAATGCCTCGCCATTAAACATTGCCT
H2170A-For	TGTTTAATGTTAAGGGCGTATCTCGAGTCAACT
H2170A-Rev	AGTTGACTCGAGATACGCCCTTAACATTAAACA
Y2171A-For	TTAATGTTAAGGCATGCGCTCGAGTCAACTGCC
Y2171A-Rev	GGCAGTTGACTCGAGCGCATGCCTTAACATTAA
H2190A-For	TATTTTAAGTATATTGCGAATCAAGAGACTAGA
H2190A-Rev	TCTAGTCTCTTGATTCGCAATATACTTAAAATA
D2225A-For	GGAGAGGAGTCGTTTGCGAGTAATATCGTGCTT
D2225A-Rev	AAGCACGATATTACTCGCAAACGACTCCTCTCC
N2227A-For	GAGTCATTTGATAGCGCGATCGTGCTTGTGACT
N2227A-Rev	AGTCACAAGCACGATCGCGCTATCAAATGACTC
I2283A-For	AAAACTCCAATAAGCGCGAACGCTGATGGTTTG
I2283A-Rev	CAAACCATCAGCGTTCGCGCTTATTGGAGTTTT
L2288A-For	ATCAACGCTGATGGTGCGTACGAGGTTATACTT
L2288A-Rev	AAGTATAACCTCGTACGCACCATCAGCGTTGAT
V2291A-For	GATGGTTTGTACGAGGCGATACTTCAAGGAGTA
V2291A-Rev	TACTCCTTGAAGTATCGCCTCGTACAAACCATC
Y2299A-For	CAAGGAGTATATACTGCGCCATACCATGGCGAT
Y2299A-Rev	ATCGCCATGGTATGGCGCAGTATATACTCCTTG
H2302A-For	TATACTTATCCATACGCGGGCGATGGTGTTTGT
H2302A-Rev	ACAAACACCATCGCCCGCGTATGGATAAGTATA
D2304A-For	TATCCATACCATGGCGCGGGTGTTTGTGGTTCG
D2304A-Rev	CGAACCACAAACACCCGCGCCATGGTATGGATA
C2307A-For	CACGGCGATGGTGTTGCGGGTTCGATATTGTTG

C2307A-Rev	CAACAATATCGAACCCGCAACACCATCGCCGTG
H2324A-For	TCAGTACCAGCAACAGCGATACCTATAATTGG
H2324A-Rev	CCAATTATAGGTATCGCTGTTGCTGGTACTGA
E2329A-For	CATGTTGCTGGTACTGCGGGATTGCATGGCTTT
E2329A-Rev	AAAGCCATGCAATCCCGCAGTACCAGCAACATG
Q2118A-For	GTGACTACTAAGCCTGCGGGATCAACACAACAA
Q2118A-Rev	TTGTTGTGTTGATCCCGCAGGCTTAGTAGTCAC
E2180A-For	ACTGCCGCCTTTCCTGCGGGAACCAAGTACTAT
E2180A-Rev	ATAGTACTTGGTTCCCGCAGGAAAGGCGGCAGT
